# Adults’ cochlear implant journeys through care: a qualitative study

**DOI:** 10.1186/s12913-020-05334-y

**Published:** 2020-05-24

**Authors:** Frances Rapport, Sarah E. Hughes, Isabelle Boisvert, Catherine M. McMahon, Jeffrey Braithwaite, Mona Faris, Mia Bierbaum

**Affiliations:** 1 Australian Institute of Health Innovation, Macquarie University, Macquarie Park, NSW Australia; 2 Swansea University Medical School, Swansea University, Swansea, Wales, United Kingdom; 3 South Wales Cochlear Implant Programme, Princess of Wales Hospital, Bridgend, Mid Glamorgan, Wales United Kingdom; 4 H:EAR [Hearing: Education, Application, Research], Australian Hearing Hub, Macquarie University, Macquarie Park, NSW Australia; 5 The HEARing Cooperative Research Centre, Melbourne, VIC Australia

**Keywords:** Sensorineural hearing loss, Cochlear implants, Hearing aids, Quality of life, Professional practice, Audiologists, General practitioners

## Abstract

**Background:**

Cochlear implants (CIs) can provide a sound sensation for those with severe sensorineural hearing loss (SNHL), benefitting speech understanding and quality of life. Nevertheless, rates of implantation remain low, and limited research investigates journeys from traditional hearing aids to implantable devices.

**Method:**

Fifty-five adults (≥ 50 years), hearing aid users and/or CI users, General Practitioners, and Australian and United Kingdom audiologists took part in a multi-methods study. Focus groups, interviews, and surveys were thematically analysed.

**Results:**

One hundred forty-three data-capture events disclosed 2 themes: 1) “*The burden of hearing loss and the impact of Cochlear Implants*”, and 2) “*Professional Support and Practice, and HCPs Roles and Responsibilities”*.

**Conclusions:**

Care experience can include convoluted, complex journeys towards cochlear implantation. The significant impact of this, as hearing loss progresses, motivates people to consider implants, but they and healthcare professionals need clear supported with defined referral pathways, and less system complexity.

## Background

The incidence of significant, acquired hearing loss in adults is rising year on year. In the United Kingdom (UK), it is estimated that 5.3 million adults over the age of 65 have a hearing loss [1] and that, by 2030, hearing loss will be in the top ten disease burdens above diabetes and cataracts [2]. In Australia, it is projected that by 2020 there will be more than 573,000 adults with severe sensorineural hearing loss (SNHL) [2]. The rising incidence of hearing loss in an ageing population is a significant public health concern as hearing loss is a known risk factor for dementia, disability, depression and mortality [3].

Additionally, hearing loss has significant negative implications for individuals in relation to communication, work and education, and social participation [4, 5], and affects quality of life [6]. As the incidence of severe SNHL rises, cost-effective interventions to mitigate the negative impact are needed. For individuals unable to derive benefit from acoustic hearing aids (HAs), cochlear implants (CIs) may be a useful surgical intervention [6, 7] including improved speech perception [5], reduced listening effort [8, 9] as well as improvements in psychosocial wellbeing, social adjustment and improved health-related quality of life [7, 10].

Despite the well-documented, positive benefits of cochlear implantation and the known costs of untreated hearing loss [11], utilisation of CIs each year remains low relative to prevalence. This is a trend reported in developed countries worldwide irrespective of these nations’ healthcare systems. Raine et al. [12] estimated the utilisation of CIs in the UK adult population, for example, is below 5%, whilst Sorkin [13] reported utilisation rates in the United States (US) of less than 5%. Similar utilisation rates are reported elsewhere [14]. The reasons for low utilisation rates are still not yet well understood and there appears to be limited research investigating factors contributing to CI uptake [8]. To the authors’ knowledge, few studies have explored the patient journey of adults with hearing loss in relation to cochlear implantation.

The patient journey may be defined as the experiences and processes the patient goes through during the course of a disease and its treatment [15]. To date, published studies have focussed on adults with hearing loss who go on to access acoustic hearing aids. For example, Engelund [16] used classical Grounded Theory to theorise that the journey of acquired hearing loss is a four-stage recognition process and a social psychological problem that has to be resolved before people will recognise their need to seek help. Manchaiah et al. [15] developed a seven-stage patient journey template in relation to hearing help-seeking and the IDA model based on qualitative accounts from patients and audiologists. There also appears to be limited research examining the views and experiences of adults with severe SNHL as they consult with community healthcare professionals (HCPs) (e.g. GPs and audiologists) before accessing specialist CI services. To the authors knowledge, no studies have been published that explore the hearing healthcare roles and responsibilities of professionals who practice outside of the CI multidisciplinary team. This is despite these clinicians having responsibility for referrals to tertiary-level CI programmes [17].

Given the limited research investigating the patient journey for adults who go on to receive CIs, this study aimed to develop a rich, in-depth understanding of the factors that impact and influence patients’ and professionals’ experiences within the context of this journey. The current study is novel in its international approach to exploring older adults’ lived experiences of hearing loss and hearing healthcare, together with its focus on professional roles and responsibilities and the complex decision-making associated with CI referral. This study also uniquely includes first-hand accounts from non-CI specialists.

### Study aims and objectives

The study explored the hearing healthcare experiences of older adults with severe (or ‘greater’) SNHL in the context of their journey to cochlear implantation. Specifically, this study sought to: 1) describe the experiences of hearing healthcare provision from the perspective of patients, GPs and audiologists and 2) gain insights into this client group’s perceived hearing healthcare needs as they investigate different treatment options including cochlear implantation.

## Methods

### Ethics approval

Ethical approval was obtained for this study from Macquarie University Human Research Ethics Committee before any data collection took place (approval no. 5201700539).

### Study design and context

This was a multi-phase, multi-method study conducted in Australia and the United Kingdom, between June 2017 and April 2018. The UK’s publicly-funded National Health Service (NHS) offers universal healthcare that is free to all legal residents from point of use [18] whereas healthcare in Australia is delivered as a mixed system of public providers and services funded by private insurance [19]. Conducting the study across different health systems enabled the study team to capture the views of different populations towards cochlear implantation and in different healthcare contexts, providing insights into specific cultural and contextual factors that may be at play [20].

### Recruitment

Promotional flyers were distributed across Australian audiology clinics, GP clinics and hearing associations, in hard copy and online, and via professional organisation websites in the UK [8]. In Australia, hearing aid (HA) users and CI users were recruited, along with audiologists, and GPs. A comparator Audiologist cohort were recruited in England and Wales (Table 1) to add rich, insightful information to add a new perspective to the Australian cohort data. Further detail regarding the methods can be found in the study protocol [22] and the parallel results paper reporting findings regarding the barriers and facilitators to CI uptake [21].

**Table 1:** Data capture events [21]

Data capture events	Australia						UK
	HA Au (*n* = 9)	CI Au (*N* = 2)	GPs (*n* = 7)	CI users (*n* = 17)	HA users (*n* = 7)	(CI cand) (*n* = 2)	HA Au (*n* = 11)
**Pilot Focus groups**	1 (*n* = 3)			1 (*n* = 5)			1 (*n* = 2)
**Pilot Interviews**			2 (*n* = 2)				
**Focus Groups**							1 (*n* = 5) 1 (*n* = 2)
**Interviews** **Face to face**							2 (*n* = 2)
**Interviews - Telephone**	6 (*n* = 6)	1 (*n* = 1)	5 (*n* = 5)	4 (*n* = 4)	3 (*n* = 3)	1 (*n* = 1)	
**Interviews - Teleconference**				1 (*n* = 1)			
**Interviews –Email**		1 (*n* = 1)		7 (*n* = 7)	4 (*n* = 4)	1 (*n* = 1)	
**Questionnaire**	8	2	7	17	7	2	11
**Survey**	7	1	6	15	6	2	9

Note: HA Au (HA Audiologist); CI Au (CI Audiologist); GP (General practitioner); CI cand (CI candidate)

#### Patient inclusion criteria

(1) 50 years of age and older, (2) with severe, post-lingual SNHL, (3) self-identified as proficient English speakers, (4) willing to participate in focus groups or interviews, and (5) willing to complete a demographic questionnaire and an open-ended qualitative survey.

#### HCP inclusion criteria

(1) GPs or audiologists HCPs, (2) working with the target patient populations, (3) willing to participate in focus groups or interviews, and (4) willing to complete a demographic questionnaire and an open-ended qualitative survey.

### Sampling and ethical considerations

Purposive timeframe sampling, a tried and tested method by this team [21, 23], was conducted. Timeframe sampling ensured that the broad range of predefined cohorts were recruited within a specific period of time, and that within that period all potential participants had an equal opportunity of participating to reduce recruitment bias [24]. Self-selection also mitigated against researcher coercion. All participants provided consent, and all data were anonymised, upholding ethical principles of good data management.

### Data collection and analysis

Focus groups and individual interviews were facilitated by a researcher, one in Australia (MB, a public health researcher) and another in the UK (SH, a speech and language therapist and health services researcher), both with extensive experience conducting research, and no prior relationships with participants. For further detail on the data collection methods see the parallel publication [21]. A set of pre-defined questions was utilized to ensure consistency and coherency across UK and Australian cohorts while researchers were in regular contact throughout data collection to ensure similar approaches were upheld. Participants chose the interview format that best suited their hearing needs (face-to-face, via telephone, video conferencing, or email). A pilot phase, consisting of three focus groups and two interviews, was conducted to ensure the interview schedules were relevant and comprehensible to participants (Table 1). No amendments to the interview questions were made following these data collection events, and so pilot data were included in the final analysis. Participants also completed a demographic questionnaire and, following the interview or focus group, a qualitative, open-ended survey. The surveys were brief and exploratory with the aim of providing additional information to initial interview and focus group answers, verify findings, and confirm data saturation [8].

All focus groups and interviews lasted approximately forty-five minutes and were audio-recorded. Focus group facilitators were trained in facilitation techniques to support communication with people with hearing loss (i.e., the use of clear speech, facing the listener). Real-time sub-titles and printed questions were provided to participants before the interviews or focus groups began, for additional support [25]. The room in which the focus groups were conducted was arranged to facilitate optimal communication. Surveys were sent out several weeks after the focus groups and interviews. This staged-approach enabled the study team to conduct an initial analysis of the focus group and interview data to inform the question content of the surveys. No amendments were made to the surveys upon early analysis of interview data, and pilot data were included in the final analysis.

All audio recordings were transcribed verbatim and thematically analysed [26] along with the survey responses, to identify the main themes across datasets. Thematic analysis, aligned with the research team’s essentialist (realist) theoretical stance [26], and enabled the analysis of the experiences of participants without over-reliance on an emergent theory. Analysis was undertaken initially by hand, and then with NVivo Pro 11 software V.11, 2015. Inductive thematic analysis [27, 28] was conducted in a staged process. A coding framework was developed inductively by a primary coder who categorised themes and sub-themes as data arrived. Teamwork followed, leading to consensus-building and refinement of the thematic framework. The technique, applied extensively in other published team studies, ensured well-validated data, and added to data veracity [29] enabling triangulation of findings [30]. Subsequent datasets were coded using the inductively derived thematic framework, as a means of identifying meaningful units of text [27].

## Results

In total, 143 data-capture events took place, including five focus groups, 38 interviews, 54 demographic questionnaires and 46 qualitative surveys. Participants were recruited across Australia and the UK (*n* = 55): 17 CI users, nine HA users (including two CI candidates who had begun CI candidacy assessment), seven GPs, and 11 Australian audiologists (9 HA audiologists and 2 CI audiologists). To compare two well-established systems using different funding models, 11 audiologists were also recruited from the UK (England and Wales) (Table 1).

### Participant characteristics

#### Patients

Most CI users reported being unilateral CI users (*n* = 13), fitted with a CI in one ear and a HA in the other (n = 8), who had used a CI for more than 3 years (*n* = 17), and were HA users for more than five years prior to implantation (*n* = 16). Many CI users (*n* = 8) travelled to access hearing services (up to 1700 km per round trip). The HA users (including two CI candidates), were all bilateral HA users (*N* = 9), and had been for more than 5 years (*n* = 9). Most HA users accessed local hearing services (*n* = 7) [8]. Greater detail published in parallel paper [21].

#### Healthcare professionals (HCPs)

Seven GP participants were recruited in Australia, most with experience of practicing for more than 10 years (*n* = 5). In Australia, 11 audiologists were recruited (10 completed the demographic questionnaire) who identified as either HA audiologists (*n* = 8) or CI audiologists (*n* = 2). Most had practiced for over 10 years (*n* = 8), in metropolitan areas (*n* = 5), as public providers (*n* = 3), private providers (*n* = 2), or both (*n* = 5). Eleven audiologists were recruited in the UK, half of whom had been practicing for 10 years or more (*n* = 6) and were either public providers (*n* = 10), or both public and private providers (*n* = 1).

### Thematic overview

The thematic framework is presented below in narrative format. Direct participant quotes are presented in italics. The thematic framework was derived from patient and HCP accounts captured in the focus group and interview transcripts as well as survey responses. Team analysis of the follow-up qualitative, open-ended survey confirmed saturation of the data, by identifying no new findings, and verified findings. Qualitative analysis of these data sets identified themes relating to barriers and facilitators to CI uptake, which have been reported elsewhere [21], as well as factors relating to the patient journey to cochlear implantation, the focus of this publication. The following section will present two overarching themes: “*The burden of hearing loss and the impact of Cochlear Implants*”, and “*Professional Support and Practice, and HCPs Roles and Responsibilities”*.

### Key themes

#### Theme 1: the burden of hearing loss and the impact of Cochlear implants

HA and CI users emphasised the impact of hearing loss on their ability to communicate effectively: “*It’s difficult for people to communicate, to engage … it halts or stops your spontaneous communication”* (HA user 1). Patients found this particularly challenging in group situations, or on the phone, as well as when trying to hear announcements on public transport, or speak to doctors and other professionals: “*It truly is soul destroying having to ask people to repeat themselves constantly” (*CI user 5). Patients described the listening effort required in these circumstances as particularly exhausting:

*I’m used to analysing things and looking for meaning beneath the surface ... Now I’m just struggling for the superficial of what are they’re saying and to me that’s been the biggest loss ...*

—CI user 4.

The effect of increased listening effort resulted in changes to HA and CI users’ sense of self-identity, and the sense that others perceived them as “*stupid because [they] can’t hear properly*” (CI user 7). They felt less sure of themselves, their self-confidence progressively declined, and they saw themselves as ineffectual communicators, who needed to find extensive adaptive behaviours:

*It’s almost like hearing impairment actually makes you inferior … People seem to be more comfortable helping someone with vision problems. They’re less patient with people who have communication difficulties.*

—HA user 1.

Some HA and CI users reported using strategies to cope, including lip reading, choosing a quiet environment to talk in, and using emails to communicate. However, this still led to frustration and stress, affecting their social life, and limiting their opportunities at work.

Most CI recipients discussed the effect of CIs on their wellbeing and were generally positive, reporting a marked improvement in their emotional wellbeing, hearing, and communication as well as reduced listening effort and listening-related fatigue: “*I would suffer from dreadful tiredness from trying to communicate all day. It used to take so much out of me to listen. Now I forget how much trouble I had and how tired and depressed it used to make me feel”* (CI user 2)*.* Many also reported that CIs improved their ability to use the phone and hear music, as well as increasing their engagement and confidence, and improving relationships with friends and family.

HA and CI users suggested improvements need to be made to professional education, to better support adults to understand their options, enabling patients to receive better information early on, and providing greater access to CI services. Similarly, they talked about the need for health promotion campaigns to support greater awareness, understanding and empathy in the general public about what it means to have a hearing loss, the impacts and limitations of CIs and HAs and the need for improved support infrastructure, and services.

*People in the everyday world, which includes health professionals, [need] to develop more understanding of hearing impairment, and not consider a [HA] or a [CI] as something that restores hearing to normal function.*

—CI user 11.

The provision of psychosocial support from hearing services, increased access to CIs for individuals, and continued financial and technological support from CI manufacturers, insurance companies and hearing organisations were seen as ways to ease the burden of hearing loss by both CI and HA users: “*I would say the only thing missing at the beginning of my discovery of my hearing impairment was emotional support to cope with hearing impairment”* (CI user 2).

Unilateral CI recipients were protective of their remaining hearing in the other ear, fearing further damage, and guarded against this by avoiding noisy situations. They were satisfied and grateful for the improvements in hearing, however a number of challenges remained, including trying to hear conversations in large groups, and using the telephone. Other issues included the need for regular battery changes, the dissatisfaction with the quality of the CI sound, and the need for safety devices, to provide alerts when they took off the external part of their device. Live entertainment also proved difficult for both HA users and CI recipients who, limited by their hearing loss, had to rely on captioning or subtitles. Some CI and HA users talked about the burden of costly devices and upgrades, problems of travelling to hearing health services, and ongoing associated maintenance costs leading to a reliance on financial support from private health insurers, and government schemes:

*I would say the main challenge is the distance that I have to go to get mappings done, to get replacements, things like that.*

—CI user 3.

#### Theme 2: professional support and practice, and HCPs roles and responsibilities

HA and CI users discussed the practical assistance they received from HCPs. Both groups found accessing health information themselves challenging, due to the severity of their hearing loss:

*Just being deaf does make it difficult to communicate with health specialists … heaven knows how I ever communicated with my doctor in the past about important health issues.*

—CI user 2.

To complement the patient perspective, HCPs discussed their practice, roles and responsibilities. CIs are first introduced by HA audiologists when patients report inadequate HA benefit, and when audiometric results suggest patients may benefit from a CI. Feeling supported and provided with consistent information was seen to be important to help patients to come to terms with changes in their hearing loss and needs. However, Australian and UK audiologists described patients as often negative about CIs to begin with and as a result, discussions and decisions about CIs often required multiple sessions:

*It’s not something that’s achieved in one visit. It’s an idea that you set the seed and develop, feeding in information as appropriate, and get them to a position where they feel they can make a decision whether to be referred or not.*

—UK audiologist 9.

Audiologists’ perceptions of their own roles varied. Some thought they were there to provide a basic service only, while others felt patients required more active support. This included: making appointments for CI clinic assessments, providing resource materials, directing patients to hearing associations, and ensuring patients were fully informed. GPs on the other hand, saw themselves as playing a coordinating and referral role only. Key decision-points in the patient journey, as perceived by HCPs, included patients’ acknowledgment of their hearing loss, accessing hearing healthcare services, deciding to have an HA fitted, agreeing to a CI assessment, and agreeing to CI surgery.

Most patients reported being satisfied with their audiologists’ advice and support, but felt that knowledge of CIs was variable amongst HA audiologists. A lack of individual clinician-patient continuity was seen to be problematic, especially if patients had to provide their hearing history repeatedly to new clinicians along the way:

*Very hard … I see a different person almost every appointment which is quite confusing having to go over everything each time…. I feel too old sometimes to bother, sometimes too frustrated with my hearing disappearing so fast and not getting answers.*

—CI candidate 1.

Audiologists felt that poor patient-clinician continuity made the building of trust challenging, and often resulted in a lack of awareness of a patient’s hearing history. HA audiologists did not always feel confident discussing CIs with patients and fragmented care could make it more difficult for professionals to confidently raise the issue of CIs with patients, and to understand the impact that conversations with preceding clinicians had on a patient’s perception of CIs.

*I’ve seen a patient and then I’ve discussed [CIs]. Then they’ve seen somebody … Then the patient won’t come back again. I wouldn’t know what happened to them … and there’s no closure.*

—UK audiologist 3.

Communication between HCPs in Australia was reported to be inconsistent and unidirectional at times, with audiological assessment reports sent to GPs, specialists, and CI clinics, but feedback rarely received by HA audiologists, whereas communication between HCPs in the UK was reported as much more consistent. In Australia, shared care was reportedly lacking, between GPs, audiologists, and specialists:

*We find ourselves sometimes chasing up communication from medical professionals, particularly specialists. Sometimes ensuring the lines of communication are open falls to the client.*

—Australian audiologist 2.

HCPs in Australia felt the referral pathways were convoluted and lacking in clarity. Whereas in the UK, audiologists reported typically sending CI candidates to either a senior audiologist or a CI clinic, as well as to hearing associations for information. Co-location of some hearing clinics with CI clinics in the UK, made bi-directional communication and shared patient information more feasible. Multidisciplinary team meetings were suggested by several UK audiologists as an effective strategy to improve shared-care, where patient cases were discussed across health specialists*.*

HA and CI users felt most GPs prioritised other health conditions over hearing health: “*The GP is really not interested in my hearing. He’s more into keeping me alive I suppose”* (CI user 6). GPs self-identified as lacking knowledge about CIs and hearing loss devices in general and felt unable to directly advise patients. They were often seen by patients as lacking in knowledge regarding hearing pathways, adding unnecessary time, especially during early-stage investigations, to the patient journey through the system.

Many HA and CI users found support groups helpful and valued attending functions where they could access CI information, and increase opportunities for social support, enabling HA participants to meet CI recipients, and gain first-hand information about CIs.

## Discussion

This multi-method study used thematic analysis, from an essentialist perspective, to provide insights into factors that impact the patient journey for adults with hearing loss, who go on to access CIs. The findings present accounts from both patients and HCPs in Australia, and audiologists in the UK. While the content of the two themes confirms previously published literature on the impact of severe SNHL and the benefits of CIs, it also contributes novel findings relating to the patient journey, especially regarding events occurring before referral to specialist CI services, and adds rich content to clarify the team’s research in this field [21, 22]. Theme content revealed underlying frustrations for both patients and professionals that resulted from lack of communication across services, limitations in shared-care, and general healthcare system complexity. Importantly, this study expands on existing theoretical frameworks relating to the patient journey for adults with hearing impairment [15] by providing insights into the care continuum, specifically in the context of cochlear implantation.

The factors that characterise the patient journey to cochlear implantation may be anchored in Manchaiah et al.’s [15] account of the patient journey for adults with hearing impairment. When viewed within this model, the experiences of the “burden of hearing loss” mined from the participant accounts are analogous to the pre-awareness and awareness stages of Manchaiah et al.’s model. The awareness and pre-awareness stages, with a SNHL, take place early on in the patient journey through assessment and care. At these stages, patients search for information, and support, and involve family and friends in conversations about their health and wellbeing, while examining past hearing experience and working out how to deal with the debilitating symptoms of hearing loss. Widespread health promotion around SNHL, and increased public awareness about what the burden of hearing loss means, is key to patient support. In addition, HCP recognition of the existence of key decision-points (such as initiating a conversation about CI referral) is representative of the “movement” stage for patients, once they recognise that their hearing will not return, and make the decision to seek help. Patient aspirations for the future improvements (i.e., better CI technology and improved support) may be situated in the “resolution” stage, when problems relating to hearing health are satisfactorily resolved and new problems may be identified.

This study provides valuable insights into the role of the non-specialist professional (i.e., GPs and HA audiologists, who sit outside of the CI multi-disciplinary team) in patient care. Few studies have explored qualitatively the care pathway up to the point of CI referral. The findings emphasise the important role that non-specialist professionals assume in this process. The patient and professional accounts support and enhance findings of a UK survey-based pilot study [31] undertaken to investigate audiologists’ knowledge of CIs and their related referrals to the CI centre. Limitations regarding professional knowledge of CIs and confidence, were identified as factors that influence the patient journey towards implantation, findings which are further supported by the work of Cohen et al. [32] The present study further extends these findings by documenting patient and professional aspirations for improved support, increased CI education for professional development, and greater access to referral tools that have been developed in conjunction with CI specialist teams.

Reduced wellbeing was reported by participants as a “burden of hearing loss” and is consistent with the established literature on psychosocial wellbeing in hearing loss [18, 19] and the benefits of CIs. We build on this literature, by highlighting reduced wellbeing as an influential factor that informs patient journeys towards implantation.

The results from this study have the potential to inform clinical practice with respect to public health. Our interpretation of participant accounts suggests that strategies to increase public awareness of hearing loss could empower patients to raise cochlear implantation with their HCPs, leading to timely referral to CI specialist programmes. Moreover, increased public awareness could empower patients’ help-seeking behaviours, particularly with regards to information access. Adult hearing loss Associations of are powerful providers of support and buddying schemes [12]. These may be particularly helpful when patients face difficult decisions about CI referral and surgery. While schemes are currently available in many countries, including Australia and the UK, the study findings suggest patients value access to expert patients and CI mentors early in their CI journey and at key points (e.g. during referral for assessment of CI candidacy). The findings also suggest that, post-implantation, CI recipients are motivated to help others in their journey to cochlear implantation and, in return, gain a sense of empowerment. These findings add to previous research exploring the positive role of support groups for CI users [33] and are compatible with previous studies investigating the effect of group support on wellbeing and social isolation [7, 34].

### Strengths and limitations

Despite the purposive timeframe sampling approach, recruitment took a lengthy period of time as HCP participants were slow to recruit. Furthermore, participant self-selection may have influenced the representativeness of the sample, which in turn has implications for the translation of these data to wider population groups. However, self-selection within a given time-period and across a wide professional group, in addition to the patient demographic-mix, meant rich data were collected from participants willing and able to offer detailed accounts of their needs and of professional support. We also note that a considerable proportion of patients were recruited via patient associations, which may have influenced the positive report these participants had towards such organisations.

The present study utilised the accounts of audiologists in the UK to examine the patient journey to cochlear implantation within a different healthcare system. The UK audiologist accounts supported findings from Australian audiologists, irrespective of the differences in service delivery and are a strength of this study. However, for pragmatic reasons, a UK sample of adults with HL who used either HAs or CIs was not included; therefore, a comparative patient perspective of the CI journey was not provided. Future research to verify and extend the findings from patient groups across different healthcare systems would further enrich study findings. In addition, future research will include the perspectives of other HCPs involved in the pathway to CIs, such as Ear Nose and Throat specialists, to develop a more comprehensive understanding of all of the services involved in the patient journey.

## Conclusions

A better understanding of the patient journey to cochlear implantation is identified as an important component of continued quality improvement in hearing healthcare. This multi-method study presented key factors that influence the CI journey for adults with severe SNHL. In identifying these factors, the study offers a picture of the hearing health experiences of Australian CI and HA users, and their reflections on professional practice, supported by the views and experiences of Australian GPs and audiologists, and corroborated by accounts from audiologists in England and Wales.

These findings suggest that patients require early, easy access to reliable information on CIs and timely referral to specialist CI services, and targeted support for patients and professionals is required at earlier stages of the CI journey. Non-specialist hearing HCPs must be confident and well-informed, and should have access to targeted, evidence-based continuing professional education and resources. Peer-support is an important element of patient care, with the aim of increasing access to reliable information and uptake of appropriate CI referrals and to ease the patient’s transition from HAs to CIs.

These strategies along with improved shared care, and better infrastructure and support strategies, have the potential to improve the QoL of adults with severe SNHL, who currently experience difficulties with their HAs, by providing more comprehensive information and timely referral to CI specialist teams. Importantly, future research should extend the study’s findings by seeking to determine the most effective strategies for CI uptake. Importantly, the patient journey continues beyond the act of receiving a CI, therefore further research is needed to extend the study’s findings, mapping the patient journey in-depth, as they move towards cochlear implantation.

## Data Availability

The data that support the findings of this study are available from the corresponding author, Frances Rapport, upon reasonable request.

## Abbreviations

CI: Cochlear Implant
ENT: Ear Nose and Throat
GP: General Practitioner
HA: Hearing Aid
HCP: Healthcare Professional
MDT: Multidisciplinary Team
NHS: National Health Service
QoL: Quality of Life
SNHL: Sensorineural Hearing Loss
UK: United Kingdom
